# Proteomic study on gender differences in aging kidney of mice

**DOI:** 10.1186/1477-5956-7-16

**Published:** 2009-04-09

**Authors:** Hanna Amelina, Susana Cristobal

**Affiliations:** 1 Department of Biochemistry and Biophysics, Stockholm University, SE-106 91, Stockholm, Sweden

## Abstract

**Background:**

This study aims to analyze sex differences in mice aging kidney. We applied a proteomic technique based on subfractionation, and liquid chromatography coupled with 2-DE. Samples from male and female CD1-Swiss outbred mice from 28 weeks, 52 weeks, and 76 weeks were analysed by 2-DE, and selected proteins were identified by matrix assisted laser desorption ionisation time-of-flight mass spectrometry (MALDI-TOF MS).

**Results:**

This proteomic analysis detected age-related changes in protein expression in 55 protein-spots, corresponding to 22 spots in males and 33 spots in females. We found a protein expression signature (PES) of aging composed by 8 spots, common for both genders. The identified proteins indicated increases in oxidative and proteolytic proteins and decreases in glycolytic proteins, and antioxidant enzymes.

**Conclusion:**

Our results provide insights into the gender differences associated to the decline of kidney function in aging. Thus, we show that proteomics can provide valuable information on age-related changes in expression levels of proteins and related modifications. This pilot study is still far from providing candidates for aging-biomarkers. However, we suggest that the analysis of these proteins could suggest mechanisms of cellular aging in kidney, and improve the kidney selection for transplantation.

## Background

Aging studies in tissues such as brain have attracted a lot of attention, however the kidney has been neglected [1]. Very recently, differential expression of proteins involved in metabolism, transport, and stress response in kidney has been reported from aging male mouse [2]. Although this organ shows a quantifiable decline of function with age, the gender differences have not been analyzed in previous proteomics studies [3]. There is an approximately 25% decline in the glomerular filtration rate starting at age 40 for humans and the ability of the medulla to concentrate urine declines progressively with age. Therefore, any disease affecting the organ, including hypertension and diabetes mellitus, accelerates the age-related changes in kidney. Moreover, impaired kidneys are targets for transplantation. Therefore, novel aging kidney biomarkers could also improve the selection of older donor organs for transplantation.

Aging is among the most complex biological phenomena. It is a complex process resulting from changes in the expression and regulation of numerous genes over time. Most physiological functions decline with age because cells accumulate damage over time. This slow incremental damage results in the gradual loss of differentiated functions and growth rate. This process is accompanied by an increased probability for the development of cancer [4]. Mounting evidences indicate that a specific gene could be connected to the extended longevity. However, the universal explanation for these life-extending effects has not yet been found. Alterations in the expression of individual proteins have reported this effect. These mechanisms include: (i) telomere repair [5]; (ii) stress response [6]; (iii) anti-oxidant defense [7]; (iv) nicotinamide deamination [8]; (v) insulin/insulin-like growth factor-1 signaling [9]; and (vi) histone deacetylation [10]. However, the global view of aging has become more complex with the understanding that some of these pathways can be connected. The ability to survey the entire proteome or a subset of the proteomes offers new opportunities to study the complex biological phenomenon of aging in an unbiased manner.

Studies in model organisms such as *Saccharomyces cerevisiae*, *Caenorhabditis elegans*, *Mus musculus*, and *Drosophila melanogaster *have provided much of our insight into the underlying biological pathways associated with aging. However, a key question is still whether the mechanisms of aging are conserved between species with different lifespan. Murine models have been used to investigate the expression of proteins and their oxidation in the brains of the senescence-accelerated mouse (SAM) as a potential animal model of Alzheimer's disease [11]; the differential expression of the liver proteome [12]; and the differential gene expression profiles in the hippocampus to reveal the mechanisms involved in age-related learning and memory deficits [13]. The CD1-Swiss outbred mouse has been utilized to study brain mitochondrial dysfunction in aging [14]. Among others, proteomic techniques have been applied to examine the effect of anti-aging agents on human endothelial cells [15], to study differential protein expression and glycosylation of membrane proteins using Hutchinson-Gilford progeria syndrome fibroblasts [16], and to investigate age-related changes in the glycation of human aortic elastin [17]. These studies clearly indicate the value of additional proteomic studies of aging.

Tissue-specific quantitative assessment of protein expression could reveal preferential biochemical pathways affected by aging. Different mammalian tissues have distinct energy needs, primary functions, and regeneration capacities. The first quantitative proteomic study of rat mitochondria from various tissues has been recently published [18]. We have applied proteomics to characterise the mouse peroxisomes from liver and kidney [19]. Comparative proteomics has been utilised to examine the effect of aging on the cellular proteome from rat skeletal muscle [20], mice brain [11], and on specific organelles such as the Golgi apparatus and endoplasmic reticulum [21] or mitochondrial proteins in mice [22], in rat [3], in bovine heart [23], and rat brain [24]. Our group has performed a peroxisomal proteomic analysis of liver and kidney in young and old mice [25].

In this study, we present a subproteomic analysis of mice kidney during the aging process focusing on the gender differences. Here, we show that although age-associated changes are widespread among different functional classes of proteins, the gender effect should not be underestimated as a differential factor in aging studies. Finally, we discuss the possible role of these age-related protein modifications in the functional lifespan of the mouse kidney.

## Results

### Proteins differentially expressed with age and gender

The principal aim of this study was to characterize age-dependent changes in the subproteome of mice kidney of both genders. The tissues were first subjected to a simple fractionation in order to obtain organelle-enriched fractions [26]. The reproducibility of fractionation procedure as well as enrichment of specific organellar fractions during purification process was controlled by enzymatic analyses and Western immunoblots [see additional files 1 and 2]. Although cell fractionation from fresh tissues could provide higher recovery, in our experiment we utilized rapidly fresh frozen kidneys in order to be able to process all the samples simultaneously. Obtained fractions were then processed by liquid chromatography using Q-sepharose as an anion exchanger. The elution fractions were applied onto 2-DE, followed by colloidal Coomassie Blue staining. On average, 300 spots were detected on each 2-DE map. Statistical analysis was utilized to compare the average spot ratio of expression between the 2-DE maps from different genders and ages.

**Figure 1:**
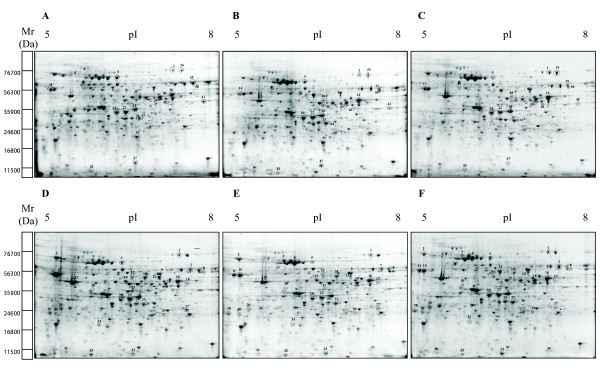
**2-DE reference gel images of male and female mouse kidney specimens from different ages showing protein expression profiles**. The equal amounts of solubilised proteins (300 μg) were separated on 11 cm pI 5-8 linear gradient strips in the first dimension and on 12.5% SDS-PAGE in the second dimension. Gels were calibrated for molecular mass (in kDa) and pI (in pI units) with external pI and mass standards and stained with CBB G-250. Proteins with significantly different expression levels between ages are marked with circles and numbered. Numbers refer to protein spot numbers in other figures and additional file 5. **(A) **gender: male, age: 28 weeks; **(B) **gender: male, age: 52 weeks; **(C) **gender: male, age: 76 weeks, **(D) **gender: female, age: 28 weeks; **(E) **gender: female, age: 52 weeks; **(F) **gender: female, age: 76 weeks.

**Figure 2:**
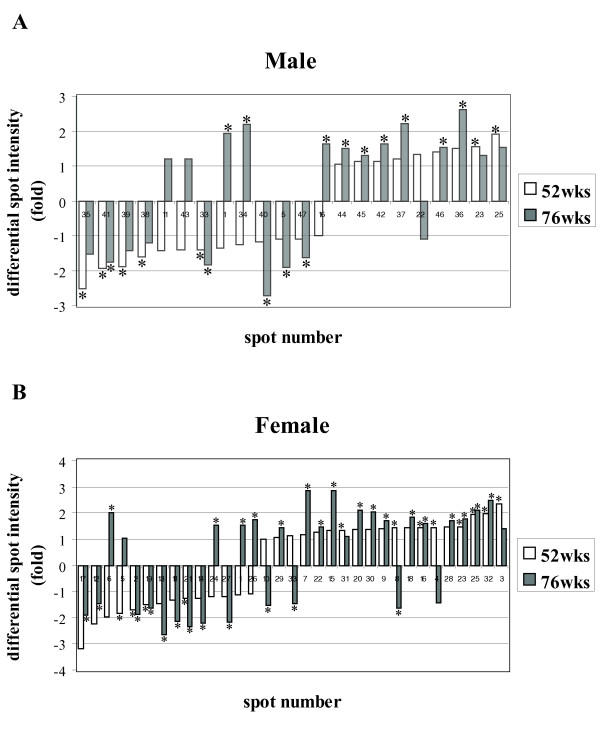
**Proteins differently expressed at the age of 52 and 76 weeks versus 28 weeks**. **(A) **male, **(B) **female. The vertical axis corresponds to differential spot intensity, above the 0 value for the up-regulated protein-spots and below the 0 value for the down-regulated ones. According to 28 weeks old group, in the horizontal axis the down-regulated proteins are organised with the lowest values on the left side and the up-regulated ones show the highest values on the right side. Statistically significant folds (one-way ANOVA, *p *< 0.05) are indicated with asterixes.

For the convenience of time-point comparisons, we divided the mice into the following groups: young mice (28 weeks old), adult mice (52 weeks old), and old mice (76 weeks old). Summarising the results, eight spots were found that compose a protein expression signature (PES) of aging, common for both genders [see additional file 3]. Studying the variation in protein expression by gender, in males, 22 protein spots showed significant changes in protein expression between young and the two groups of older mouse tissues. In the 52-week old group, 13 spots were down-regulated up to 2.5 fold, and 9 spots were up-regulated up to 1.9-fold. In the 76-week old group, 8 spots were down-regulated up to 2.7-fold and 13 spots were up-regulated up to 2.6-fold. These differentially expressed proteins are illustrated in Fig. 1A, 1B, 1C, 2A and additional file 4.

In females, 33 spots showed significant differences in protein expression between young and the two groups of older mouse tissues. In the 52-week-old group, 14 spots were down-regulated up to 3.2-fold, and 19 spots were up-regulated up to 2.4-fold. In the 76 week-old group, 13 spots were down-regulated up to 2.6-fold, and 20 spots were up-regulated up to 2.8-fold (Fig. 1D, 1E, 1F and 2B). Detailed protein expression data can be found in additional file 4.

### Multivariate analysis

**Figure 3:**
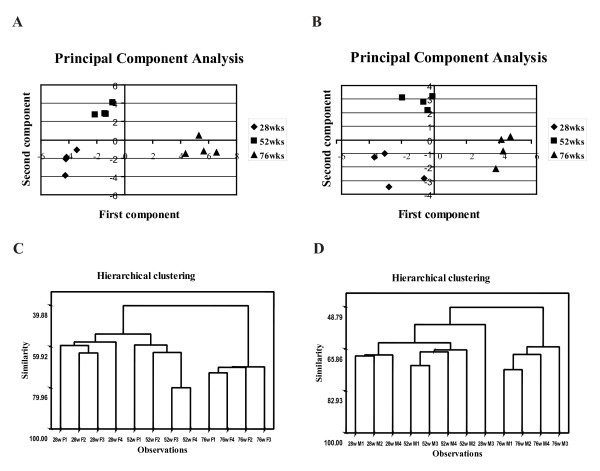
**Organization of data by multivariate analysis**. **(A) **and **(C**) correspond to female; **(B) **and **(D) **correspond to male. **(A) **and **(B) **– principle component analysis score plots. PCA has been performed on a correlation matrix, each spot represents a gel. **(C) **and **(D) **– hierarchical clustering dendrogram with single linkage and Euclidean distance. The spots that participated in both types of multivariate analysis were present in 100% of the 2-DE maps and passed the filter of the one-way ANOVA (p < 0.05) statistical test.

To validate whether the obtained PES could constitute a robust set of biomarkers, principal component analysis (PCA) and hierarchical clustering were performed. PCA is a useful tool for data categorization, since it separates the dominating features in a data set. It is remarkable that the first principal component clearly discriminates the oldest group from the two younger groups in both genders. Both sets of gels, corresponding to males and females, are situated in the positive side of the x-axis (Fig. 3A and 3B). The second principal component separated adult groups from the young ones. The adult group is clustered in the positive side of the y-axis, opposite from the young group.

Hierarchical clustering supported the results obtained with PCA. All of the gels were clustered together with a 39% similarity for female samples and 48% similarity for the male samples. For both genders, the similarity between young and adults was over 50% and separated those two groups from the old samples. The group with the highest similarity among all groups was the oldest female specimens, which showed 70% similarity level (Fig. 3C and 3D).

### Identification of differentially expressed proteins

**Figure 4:**
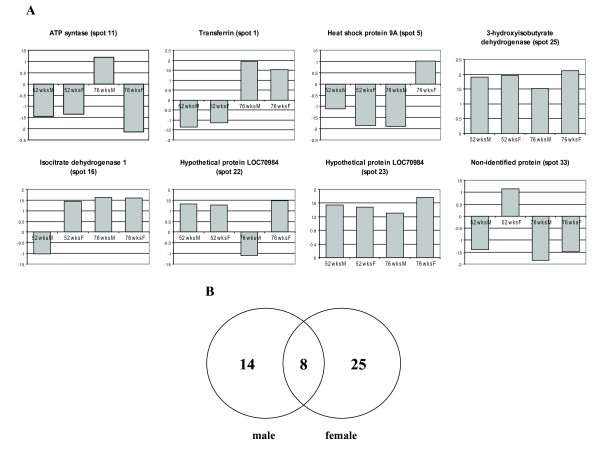
**Protein expression signature common for male and female aged mouse kidney**. **(A) **Differential protein expression in fold from the 8 proteins composing common age-related protein expression profiles in both genders. **(B) **Venn diagram representing differently expressed proteins in common for male and female mouse kidney.

The proteins that showed significant age-dependent variation in expression from male and female mouse kidney were trypsin-digested and analysed by MALDI-TOF MS. For males, 12 proteins, and 23 proteins for females, were successfully identified [see additional file 5] These spots composing an aging-PES were identified in this study (Fig. 1 and 4). These proteins are involved in energetic pathways and transport, and one of them (heat shock protein 9A) functions as molecular chaperone. We can organise the aging-related response in the following patterns of expression: a group of proteins that showed the same tendency of response in both genders, and a group of proteins that responded in a different manner depending on the gender. A group of proteins that were up-regulated in both genders is composed of transferrin, 3- hydroxyisobutyrate dehydrogenase, isocytrate dehydrogenase 1, and the hypothetical protein LOC70984 (spot 23); a group of proteins that were down-regulated in both genders includes spot-protein number 33 that we were unable to identify. A group of proteins with gender-specific response consists of ATP synthase and another isoform of the hypothetical protein LOC70984 (spot 22), that were up-regulated in males and down-regulated in females; and a group of proteins that were down-regulated in males and up-regulated in females represented by heat shock protein 9A.

### Functional classification of the differentially expressed proteins

**Figure 5:**
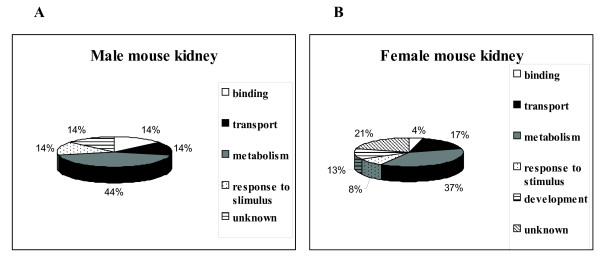
**Classification of identified age-related male (A) and female (B) mouse kidney proteins according to biological process characterization of the Gene Ontology system**.

The proteins identified in this study were functional classified by their biological association with the aging process. Using AmiGO browser  and gene onthology (GO) mapping data serviced from QickGO , the identified proteins were classified into the following groups according to their biological process: binding, transport, metabolism, response to stimulus, development, and biological process unknown. In most cases, we used GO mapping of the proteins from *Mus musculus*, and from *Rattus norvegicus *when there was no information available for the mouse proteins. We categorized 12 identified proteins from male mouse kidney and 23 proteins from female mouse kidney according to the biological process described in GO terms (Fig. 5). The functional classification showed that metabolism-associated proteins played an equally important role in the age-associated changes in protein expression for both genders, whereas the expression level of proteins involved in the developmental processes changed significantly with age only in the case of females.

## Discussion

### Proteins differentially expressed with age and gender

This first aim of this subproteomic analysis was to study sex differences in mice aging kidney and to discover possible novel biomarkers. The understanding of kidney age-related changes that compromise function of the organ during aging could improve the organ selection for kidney transplantation. In order to reduce proteome complexity and gain resolution, we utilised simple fractionation with subsequent anion-exchange chromatography coupled with 2-DE. This method was initially developed in our laboratory and successfully applied for the marine pollution assessment [26]. Using this technique, we obtained an organelle-enriched fraction and some cytosolic proteins. The reproducibility of this method and the fraction characterization was well-defined in our previous studies [26]. The fraction mainly contained mitochondria, peroxisomes, and microsomes, the organelles known to be affected by aging [21,25,27].

The aging-specific proteomic changes observed in this study highlight the importance of gender differences. Two pieces of evidences support this idea: i) the variation in protein expression with age was higher in females than in male samples; and ii) the functional classification of identified proteins revealed an important role of functions such as metabolism and response to stimulus in males and development in females. The gender difference has emerged as an important factor influencing protein expression. In aging studies, the longer female life span has been reported for humans, but gender differences have also been observed in non-human mammals [28], other vertebrates [29], and invertebrates [30]. In studies of telomere length in middle-aged populations, a significantly faster rate of age-dependent telomere-length decrease has been reported in men compared to women [31]. Oxidative damage is also an important factor in reducing life span [32] and males and females suffer different levels of oxidative damage. Both, human and mice males express lower level of protective enzymes against oxidative damage [33,34]. However, a similar age-related expression pattern was observed in both genders, consisting on an increase in the number of up-regulated proteins and a decrease in the number of down-regulated ones.

We observed that in CD1-Swiss outbred mice, a mouse model for accelerated aging [14], the ratio of aging-related proteomic changes in the studied subproteome was moderate, slightly higher for females than for male samples. However, we have previously detected stronger age-related variation from proteins involved in mitochondrial β-oxidation from kidney samples of aged male C57bl/6J mice [25], and Kim et al. [3] have also reported a decrease over 6-fold in proteins from the cytoskeleton in the rat proteome. The differences between these three studies could be attributed to utilising various species and focusing on different subproteomes. Hence, the battery of protein expression changes reported here could be explained by theories of aging. Age-specific functional decline is not a result of the complete failure of a small number of cellular processes, it is rather a slight weakening of many pathways that cumulatively causes a significant decrease in cell function [35].

The statistical analysis confirmed the robustness of the proteomic analysis method using biological replicates. The gels from young males are the only ones that showed certain interindividual variability, and the gels from the older group were the most similar, independent of gender. The proteome variations could clearly divide young and adults from the group of the old ones. In the PCA, the gels from the young and adult groups, and the gels from the old group occupied opposite sides of the first component, and the young samples were also clustered together and differentiated from the adults in the second component. Moreover, the differences between the adults and the oldest group arise from the hierarchical analysis. In agreement with this observation, a decline in protein expression at the age of 18 months and a recovery in a 24-month-old group has been reported [25].

### Identification of differentially expressed proteins

The identification of seven out of the eight proteins that composed the common PES associated with aging in this study arose some points of discussion. Among these proteins, transferrin (spot 1) was significantly down-regulated in the adult group and up-regulated in the older group in both genders. Although the main role of transferrin is to work as an iron carrier, it plays additional functions in the maintenance of metabolic activities in mammalian cells as growth, survival, and differentiation factor [36]. Therefore, changes in the expression of transferrin could be correlated with aging-associated kidney impairment. On one hand, binding of iron, which can be toxic, promotes free radical formation via the Fenton and Haber-Weiss reactions, thus resulting in oxidative damage to tissues [37]. On the other hand, decrease of transferrin levels may lead to pathological free-serum iron levels rendering individuals more susceptible to infection by iron-dependent microorganisms [38]. In addition, an age-related increase in transferrin in urine as an indirect effect of alterations of renal tubular proximal receptors with age has been reported [39].

Another up-regulated enzyme of this group was isocitrate dehydrogenase 1 (IDH). This protein provides NADPH in cytosolic and peroxisomal compartments of mammalian cells and contains a type I peroxisomal targeting sequence (an Ala-Lys-Leu tripeptide at the carboxyl terminus). The mammalian IDH fulfills many cellular functions, particularly in peroxisomes, where NADPH is required for the β-oxidation of very long-chain fatty acids and for the biosynthesis of isoprenoids, ether phospholipids, and potentially cholesterol [40]. In cell culture systems, the overexpression of IDH was correlated with protection against damage from oxidants or radiation [41,42]. Moreover, in another 2-DE-based study, this enzyme was also up-regulated in the adult rat brain [43]. The up-regulation of this protein to improve the cell protection against oxidation could partially explain the increases detected in our study. In IMR-90 cell cultures, the cytosolic IDH gradually increased with age up to the 46–48 population doubling level and then gradually decreased [44].

Age-dependent up-regulation of 3-hydroxyisobutyrate dehydrogenase, a mitochondrial enzyme involved in valine catabolism, has been previously reported. It has been shown that higher levels of 3-hydroxyisobutyrate dehydrogenase may reflect the increased need for energy because of the reduced glucose metabolism in the neural retinas of old rat [45]. On the other hand, in young tissues, tumour necrosis factor α and the peroxisome proliferator, nafenopin, induce the up-regulation of this protein in rat hepatocytes [46].

Among the proteins that showed various age-related expression pattern in different genders, the heat shock protein (HSP) 9A (spot 5) was identified. HSP 9A was down-regulated with age in males whereas it was up-regulated in females. On the contrary, ATP synthase was up-regulated in males and down-regulated in females. It has been reported that the improved efficiency of the mitochondrial respiratory chain could directly decrease the generation of ROS [47]. Two enzymes involved in the ammonia detoxication pathway showed opposite response with age in females, with up-regulation of ornithine aminotransferase and down-regulation of glutamate dehydrogenase. However, the levels of these enzymes also increased in the aged group of a senescence-accelerated mouse but not in the aged control group [12]. The possible increase in the accumulation of toxic free ammonia with age could be a consequence of this cellular response. On the other hand, the down-regulation of the glycolytic enzyme, phosphoglycerate mutase, that we observed in this study has been extensively reported for Alzheimer's disease brain samples [48]. This reduced protein expression in combination with impairment of protein function caused by its oxidation would increase the aging associated oxidative damage caused by ROS. It has also been proposed that high glycolytic rate can partially protect the cells against oxidative damage, however, oxidative stress eventually inhibits glycolysis [49].

In the framework of the male aging-specific response, down-regulation of catalase has been found. In our previous work, a similar reduction in catalase activity was observed in the kidney samples from 18-month-old male C57bl/8J mice [25]. Aging compromises the importing of peroxisomal proteins, with catalase being particularly affected. As a consequence, the relative ratio of oxidases to catalase is out of balance and could contribute to the cellular oxidative stress and aging. [50]. Finally, glutathione (GSH) levels have been shown to decrease with aging; therefore, an up-regulation of glutathione synthetase, which catalyses the second reaction in *de novo *GSH synthesis, could represent a compensatory mechanism of aging impairment. Moreover, gender differences in the concentration of GSH has been demonstrated in different tissues, with a more dramatic decrease in males [51]. These results suggest some possible molecular basis underlying the gender-associated differences in longevity.

## Conclusion

Aging kidney is still a controversial issue, several studies pointed out that certain structural dysfunctions impaired kidney with age and others suggested that the molecular age of a kidney was not directly correlated with the individuals age [52]. There is still a need to better understand the process of renal senescence, and to include the gender factor in those studies. Those are the key factors to find biomarkers of aging kidney. The outcome from our pilot study in mice is still far from providing novel candidates for biomarkers. However, the biological functions of the identified proteins revealed indirect evidence of age-related physiological changes. Therefore, we emphasize that an extensive proteomic analysis is needed to gain a better understanding of the mechanisms involved in the aging kidney, and to interpret the biological significance of the protein profiles. This approach could accelerate and lead to a high-throughput screening that could result in a better identification of organs for transplantation.

## Methods

### Animals and tissue

The mice used in this study were CD1-Swiss outbred strain of 28- (young), 52- (adult), and 76-(old) week-old, both male and female specimens [14]. The animals were grown at the Department of Experimental Animals of the University of Cadiz, housed in small groups, and kept at 22 ± 2°C with 12:12-h light-dark cycles and with full access to water and food. Experiments were carried out in accordance with the *Guiding Principles for Research Involving Animals and Human Beings *of the American Physiological Society, the Guidelines of the European Union Council (86/609/CEE), and the Spanish regulations (BOE 67/8509-12, 1988) for laboratory animals and were approved by the Scientific Committee of the University of Cadiz. The animals were euthanized by cervical dislocation, kidneys were collected, and frozen immediately and stored at -80°C until further use. The dissected frozen kidney tissues were kindly provided by Dr. A. Navarro at University of Cádiz (Spain). All mice were sacrificed the same day; four kidney samples per each age and gender group (24 kidney samples in total) were used in this study.

### Sample preparation and 2-DE PAGE

Mice kidney homogenates were prepared by previously described methods [26]. Briefly, frozen tissues (approximately 200 mg per sample) were homogenized in ice-cold homogenization buffer containing 250 mM sucrose, 5 mM MOPS, 1 mM EDTA-Na_2_, 0.1% ethanol (v/v), 0.2 mM DTT, and the following protease inhibitors: 0.2 mM PMSF, 2 μM leupeptin, 2 μM pepstatin, 1 mM ε-aminocaproic acid. The homogenates were centrifuged at 100 × *g *for 10 min at 4°C to pellet the cell debris and nuclei. The pellet was resuspended in homogenization buffer and centrifuged again at 100 × *g *for 10 min. The post-nuclear supernatants were collected and centrifuged at 1950 × *g *for 10 min, and the final supernatant was subjected to anion-exchange chromatography with Q-Sepharose Fast Flow (Amersham Biosciences, Uppsala, Sweden) as a matrix. This step reveals a larger number of low-abundance proteins and to remove proteins that otherwise would interfere with the resolution. All the steps of chromatography procedure were performed as previously described [26]. The Q-Sepharose Fast Flow matrix was equilibrated three times with 40 mM Tris-HCl buffer, pH 8.0. One volume of the organelle-enriched fraction was combined with another volume of dilution buffer, 40 mM Tris-HCl, pH 9.0, to obtain a final pH of 8.0. This fraction was loaded onto a tube containing equilibrated Q-Sepharose beads and incubated on ice for 15 min. The flow-through fractions were discarded, and Q-Sepharose beads were washed three times with 40 mM Tris-HCl, pH 8.0, and finally, it was eluted twice with elution buffer containing 40 mM Tris-HCl and 1 M KCl, pH 8.0. The eluted fractions were precipitated by 20% TCA in 100% cold acetone with 0.07% β-mercaptoethanol and washed with 1 ml acetone and 0.07% (v/v) β-mercaptoethanol. Proteins extracted by this method were solubilized in a solubilization buffer (7 M urea, 2 M thiourea, 2% CHAPS (w/v), 0.5% Triton X-100, 1% β-mercaptoethanol, 1% (v/v) Pharmalyte (3–10), 1% DTT (w/v)), modified from Rabilloud [53]. Afterwards, samples were alkylated with 30 mM IAA for 15 min in darkness and then mixed with a rehydration solution containing 8 M urea, 2% CHAPS (w/v), 15 mM DTT, 1% β-mercaptoethanol (v/v) and 0.2% Pharmalyte (v/v) (3–10). Solubilized samples were applied onto 11 cm IPG strips, pH 4–7 (Bio-Rad, Hercules, CA). Protein concentrations were measured according to Bradford [54] using bovine serum albumin as a standard. The total protein applied per gel was 300 μg. Isoelectric focusing was performed on a Protean IEF Cell (Bio-Rad) at 20°C using the following program: passive rehydration for 12 h, rapid voltage slope at all the steps, step 1: 250 V for 15 min, step 2: 8000 V for 2.5 h and step 3: at 8000 V until it reached 35000 Vh. After this, the IPG strips were reduced (1% DTT (w/v)) and then were alkylated (4% IAA (w/v)) in equilibration buffer (6 M urea, 50 mM Tris pH 8.8, 30% glycerol (v/v), 2% SDS (w/v) and 0.002% CBB (w/v)). The second dimension was carried out on homogeneous 12.5% T Criterion precast gels (Bio-Rad, Hercules, CA), at 120 V for 2 h using a Criterion Dodeca Cell (Bio-Rad).

### Image acquisition and analysis

The protein spots in the gels were visualized by staining with Coomassie Brilliant Blue G250 (CBB G250), and the gel images were obtained using Image Scanner (GE Healthcare, Uppsala, Sweden). Image Master 2D Platinum 6.0 software (GE Healthcare) was used for matching and analysis of visualized spots among differential gels and membranes to compare the level of protein expression between kidney from young, adult and old mice. Each spot intensity volume was processed by background substraction and total spot volume normalization, giving the spot volume percentage (Vol%). For the matching, two match sets were created grouping gels from different genders, four gels in each match set were organized into three match sets according to age. After completion of spot matching, the normalized spot intensity of each protein spot from individual gels was compared between groups using statistical analysis. Statistical significance was assessed by one-way ANOVA using MINITAB 14 software, with probability value *p *< 0.05 considered significant. For spots that showed significant differences, multiple comparisons were performed using Tukey test.

### Multivariate analysis

Using Image Master 2D Platinum 6.0 and MINITAB 14 statistical software, the data were processed with two different kinds of multivariate analysis: PCA and hierarchical clustering, including protein spots present in 100% of the 2-DE maps and significantly expressed according to statistical analysis.

### Trypsin digestion and MALDI-TOF MS analysis

The protein spots, excised with blade and transferred into a microcentrifuge tube, were washed 3 times with 25 mM ammonium bicarbonate (NH_4_HCO_3_) in 50% acetonitrile (ACN), followed by dehydration with 100% ACN. The solvent was then removed, and the gel pieces were dried in a flow hood. The gel pieces were then rehydrated with 12.5 ng/μl modified trypsin (Promega, Madison, WI, USA) in 50 mM NH_4_HCO_3 _with the minimal volume to cover the gel pieces and incubated overnight at 37°C. Peptides were extracted with 50% ACN/0.1% TFA, desalted, and concentrated using ZipTip_C18 _(Millipore, Billerica, MA) in accordance with manufacturer instructions. The peptide extracts (1 μl) were mixed with an equal volume of saturated matrix solution (10 mg/ml of α-cyano-4-hydroxycinnamic acid (Sigma) in 50% ACN/0.1% TFA) directly on the target and allowed to dry at room temperature. MALDI-TOF analysis was performed in reflector mode on a Voyager-DE STR MALDI-TOF mass spectrometer from Applied Biosystems (Foster City, CA). The external calibration was carried out using Sequazyme Peptide Mass Standard Kit (Applied Biosystems). Trypsin autodigestion products (842.51, 1045.56, and 2211.10 Da) were used for internal calibration. The MALDI spectra used for protein identification were searched against the National Center for Biotechnology Information (NCBI) protein databases using the MASCOT search engine . Peptide mass fingerprinting used the assumption that peptides are monoisotopic, oxidized at methionine residues, and carbamidomethylated at cysteine residues. Up to one missed trypsin cleavage was allowed. A mass tolerance of 100 ppm was the window of error allowed for matching the peptide mass values. Probability-based MOWSE scores were estimated by comparison of search results against estimated random match population and were reported as -10 × log_10_(p) where p is the absolute probability. MOWSE scores greater than 63 were considered significant. All the protein identifications were in the expected size ranges based on position on the gel. All spectra were averaged over 300 shots.

## List of abbreviations

2-DE: two-dimensional gel electrophoresis; MALDI-TOF MS: matrix assisted laser desorption ionisation time-of-flight mass spectrometry; DNPH: 2, 4-dinitrophenylhydrazine; PES: protein expression signature; HSP: heat shock protein; IDH: isocitrate dehydrogenase; GSH: gluthathione reduced; CBB G-250: Coomassie Brilliant Blue G250.

## Competing interests

The authors declare that they have no competing interests.

## Authors' contributions

HA carried out the 2-DE and MALDI-MS analysis, performed the statistical analysis, and drafted the manuscript. SC was responsible for designing the experimental strategy and writing the manuscript. HA and SC read and approved the final manuscript.

## Supplementary Material

Additional file 1 **Enrichment of some organellar marker enzymes during the subcellular fractionation procedure**. **A **– Graph illustrating the purification of acid phosphatase (lysosomal marker); **B **– graph illustrating the purification of catalase (peroxisomal marker); **C **– supplementary table with information on the absolute values of specific acid phosphatase activity at different fractionation steps; **D **– supplementary table with information on the absolute values of specific catalase activity at different fractionation steps. A – total homogenate; B – postnuclear fraction; C – heavy mitochondrial fraction; D – organelle – enriched fraction; E – eluate. Purification of an enzyme was calculated as SEA(fr.X)/SEA(fr.A), where SEA is specific enzymatic activity, and fr. X is fraction B – E. Click here for file

Additional file 2 **Representative Western blots illustrating the enrichment of specific organelles during the fractionation procedure**. A – total homogenate; B – postnuclear fraction; C – heavy mitochondrial fraction; D – organelle – enriched fraction; E – eluate from LC. Click here for file

Additional file 3 **3-D views of differentially expressed proteins common for both genders**. Click here for file

Additional file 4 **Protein expression data**. A- Female mouse kidney, B- Male mouse kidney. Click here for file

Additional file 5 **List of age-related mouse kidney proteins identified by MALDI-TOF-MS**. (A) Female; (B) Male. Proteins' numbers correspond to the numbers from 2-DE gels shown in Figs. 1 and 2. Obs., observed. Click here for file

## References

[B1] Yang S, Liu T, Li S, Zhang X, Ding Q, Que H, Yan X, Wei K, Liu S (2008). Comparative proteomic analysis of brains of naturally aging mice. Neuroscience.

[B2] Chakravarti B, Seshi B, Ratanaprayul W, Dalal N, Lin L, Raval A, Chakravarti DN (2009). Proteome profiling of aging in mouse models: differential expression of proteins involved in metabolism, transport, and stress response in kidney. Proteomics.

[B3] Kim CH, Park DU, Chung AS, Zou Y, Jung KJ, Sung BK, Yu BP, Chung HY (2004). Proteomic analysis of post-mitochondrial fractions of young and old rat kidney. Exp Gerontol.

[B4] Rubin H (1997). Cell aging in vivo and in vitro. Mech Ageing Dev.

[B5] Hemann MT, Strong MA, Hao LY, Greider CW (2001). The shortest telomere, not average telomere length, is critical for cell viability and chromosome stability. Cell.

[B6] Song J, Takeda M, Morimoto RI (2001). Bag1-Hsp70 mediates a physiological stress signalling pathway that regulates Raf-1/ERK and cell growth. Nat Cell Biol.

[B7] Melov S (2000). Mitochondrial oxidative stress. Physiologic consequences and potential for a role in aging. Ann N Y Acad Sci.

[B8] Anderson RM, Bitterman KJ, Wood JG, Medvedik O, Sinclair DA (2003). Nicotinamide and PNC1 govern lifespan extension by calorie restriction in Saccharomyces cerevisiae. Nature.

[B9] Arantes-Oliveira N, Berman JR, Kenyon C (2003). Healthy animals with extreme longevity. Science.

[B10] Tissenbaum HA, Guarente L (2001). Increased dosage of a sir-2 gene extends lifespan in Caenorhabditis elegans. Nature.

[B11] Poon HF, Vaishnav RA, Getchell TV, Getchell ML, Butterfield DA (2006). Quantitative proteomics analysis of differential protein expression and oxidative modification of specific proteins in the brains of old mice. Neurobiol Aging.

[B12] Cho YM, Bae SH, Choi BK, Cho SY, Song CW, Yoo JK, Paik YK (2003). Differential expression of the liver proteome in senescence accelerated mice. Proteomics.

[B13] Cheng XR, Zhou WX, Zhang YX, Zhou DS, Yang RF, Chen LF (2007). Differential gene expression profiles in the hippocampus of senescence-accelerated mouse. Neurobiol Aging.

[B14] Navarro A, Boveris A (2007). Brain mitochondrial dysfunction in aging: conditions that improve survival, neurological performance and mitochondrial function. Front Biosci.

[B15] Lee JH, Chung KY, Bang D, Lee KH (2006). Searching for aging-related proteins in human dermal microvascular endothelial cells treated with anti-aging agents. Proteomics.

[B16] Robinson LJ, Karlsson NG, Weiss AS, Packer NH (2003). Proteomic analysis of the genetic premature aging disease Hutchinson Gilford progeria syndrome reveals differential protein expression and glycosylation. J Proteome Res.

[B17] Konova E, Baydanoff S, Atanasova M, Velkova A (2004). Age-related changes in the glycation of human aortic elastin. Exp Gerontol.

[B18] Forner F, Foster LJ, Campanaro S, Valle G, Mann M (2006). Quantitative proteomic comparison of rat mitochondria from muscle, heart, and liver. Mol Cell Proteomics.

[B19] Mi J, Kirchner E, Cristobal S (2007). Quantitative proteomic comparison of mouse peroxisomes from liver and kidney. Proteomics.

[B20] Piec I, Listrat A, Alliot J, Chambon C, Taylor RG, Bechet D (2005). Differential proteome analysis of aging in rat skeletal muscle. Faseb J.

[B21] Drahos KL, Tran HC, Kiri AN, Lan W, McRorie DK, Horn MJ (2005). Comparison of Golgi apparatus and endoplasmic reticulum proteins from livers of juvenile and aged rats using a novel technique for separation and enrichment of organelles. J Biomol Tech.

[B22] Chang J, Van Remmen H, Cornell J, Richardson A, Ward WF (2003). Comparative proteomics: characterization of a two-dimensional gel electrophoresis system to study the effect of aging on mitochondrial proteins. Mech Ageing Dev.

[B23] Kiri AN, Tran HC, Drahos KL, Lan W, McRorie DK, Horn MJ (2005). Proteomic changes in bovine heart mitochondria with age: using a novel technique for organelle separation and enrichment. J Biomol Tech.

[B24] Poon HF, Shepherd HM, Reed TT, Calabrese V, Stella AM, Pennisi G, Cai J, Pierce WM, Klein JB, Butterfield DA (2006). Proteomics analysis provides insight into caloric restriction mediated oxidation and expression of brain proteins associated with age-related impaired cellular processes: Mitochondrial dysfunction, glutamate dysregulation and impaired protein synthesis. Neurobiol Aging.

[B25] Mi J, Garcia-Arcos I, Alvarez R, Cristobal S (2007). Age-related subproteomic analysis of mouse liver and kidney peroxisomes. Proteome Sci.

[B26] Amelina H, Apraiz I, Sun W, Cristobal S (2007). Proteomics-based method for the assessment of marine pollution using liquid chromatography coupled with two-dimensional electrophoresis. J Proteome Res.

[B27] Navarro A, Lopez-Cepero JM, Bandez MJ, Sanchez-Pino MJ, Gomez C, Cadenas E, Boveris A (2008). Hippocampal mitochondrial dysfunction in rat aging. Am J Physiol Regul Integr Comp Physiol.

[B28] Moore SL, Wilson K (2002). Parasites as a viability cost of sexual selection in natural populations of mammals. Science.

[B29] Ottinger MA, Mobarak M, Abdelnabi M, Roth G, Proudman J, Ingram DK (2005). Effects of calorie restriction on reproductive and adrenal systems in Japanese quail: are responses similar to mammals, particularly primates?. Mech Ageing Dev.

[B30] Das M (1994). Age determination and longevity in fishes. Gerontology.

[B31] Bekaert S, De Meyer T, Rietzschel ER, De Buyzere ML, De Bacquer D, Langlois M, Segers P, Cooman L, Van Damme P, Cassiman P (2007). Telomere length and cardiovascular risk factors in a middle-aged population free of overt cardiovascular disease. Aging Cell.

[B32] Johnson FB, Sinclair DA, Guarente L (1999). Molecular biology of aging. Cell.

[B33] Ide T, Tsutsui H, Ohashi N, Hayashidani S, Suematsu N, Tsuchihashi M, Tamai H, Takeshita A (2002). Greater oxidative stress in healthy young men compared with premenopausal women. Arterioscler Thromb Vasc Biol.

[B34] Tomas-Zapico C, Alvarez-Garcia O, Sierra V, Vega-Naredo I, Caballero B, Joaquin Garcia J, Acuna-Castroviejo D, Rodriguez MI, Tolivia D, Rodriguez-Colunga MJ, Coto-Montes A (2006). Oxidative damage in the livers of senescence-accelerated mice: a gender-related response. Can J Physiol Pharmacol.

[B35] Rodwell GE, Sonu R, Zahn JM, Lund J, Wilhelmy J, Wang L, Xiao W, Mindrinos M, Crane E, Segal E (2004). A transcriptional profile of aging in the human kidney. PLoS Biol.

[B36] de Jong G, van Dijk JP, van Eijk HG (1990). The biology of transferrin. Clin Chim Acta.

[B37] Kalinowski DS, Richardson DR (2005). The evolution of iron chelators for the treatment of iron overload disease and cancer. Pharmacol Rev.

[B38] Beard JL (2001). Iron biology in immune function, muscle metabolism and neuronal functioning. J Nutr.

[B39] Odera K, Goto S, Takahashi R (2007). Age-related change of endocytic receptors megalin and cubilin in the kidney in rats. Biogerontology.

[B40] Bosch H van den, Schutgens RB, Wanders RJ, Tager JM (1992). Biochemistry of peroxisomes. Annu Rev Biochem.

[B41] Kim SY, Park JW (2003). Cellular defense against singlet oxygen-induced oxidative damage by cytosolic NADP+-dependent isocitrate dehydrogenase. Free Radic Res.

[B42] Lee SM, Koh HJ, Park DC, Song BJ, Huh TL, Park JW (2002). Cytosolic NADP(+)-dependent isocitrate dehydrogenase status modulates oxidative damage to cells. Free Radic Biol Med.

[B43] Fountoulakis M, Hardmaier R, Schuller E, Lubec G (2000). Differences in protein level between neonatal and adult brain. Electrophoresis.

[B44] Kil IS, Huh TL, Lee YS, Lee YM, Park JW (2006). Regulation of replicative senescence by NADP+ -dependent isocitrate dehydrogenase. Free Radic Biol Med.

[B45] Li D, Sun F, Wang K (2004). Protein profile of aging and its retardation by caloric restriction in neural retina. Biochem Biophys Res Commun.

[B46] Chevalier S, Macdonald N, Tonge R, Rayner S, Rowlinson R, Shaw J, Young J, Davison M, Roberts RA (2000). Proteomic analysis of differential protein expression in primary hepatocytes induced by EGF, tumour necrosis factor alpha or the peroxisome proliferator nafenopin. Eur J Biochem.

[B47] Bliznakov EG (1999). Aging, mitochondria, and coenzyme Q(10): the neglected relationship. Biochimie.

[B48] Sultana R, Boyd-Kimball D, Poon HF, Cai J, Pierce WM, Klein JB, Merchant M, Markesbery WR, Butterfield DA (2006). Redox proteomics identification of oxidized proteins in Alzheimer's disease hippocampus and cerebellum: an approach to understand pathological and biochemical alterations in AD. Neurobiol Aging.

[B49] Kondoh H, Lleonart ME, Gil J, Wang J, Degan P, Peters G, Martinez D, Carnero A, Beach D (2005). Glycolytic enzymes can modulate cellular life span. Cancer Res.

[B50] Legakis JE, Koepke JI, Jedeszko C, Barlaskar F, Terlecky LJ, Edwards HJ, Walton PA, Terlecky SR (2002). Peroxisome senescence in human fibroblasts. Mol Biol Cell.

[B51] Wang H, Liu H, Liu RM (2003). Gender difference in glutathione metabolism during aging in mice. Exp Gerontol.

[B52] Tan JC, Workeneh B, Busque S, Blouch K, Derby G, Myers BD (2009). Glomerular function, structure, and number in renal allografts from older deceased donors. J Am Soc Nephrol.

[B53] Rabilloud T (1998). Use of thiourea to increase the solubility of membrane proteins in two-dimensional electrophoresis. Electrophoresis.

[B54] Bradford MM (1976). A rapid and sensitive method for the quantitation of microgram quantities of protein utilizing the principle of protein-dye binding. Anal Biochem.

