# Supplementary figures and images for: Proteomic study on gender differences in aging kidney of mice
Source: Proteome Sci. 2009 Apr 9;7:16. doi: 10.1186/1477-5956-7-16 (PMC2673210; doi:10.1186/1477-5956-7-16)

Additional file 1

A

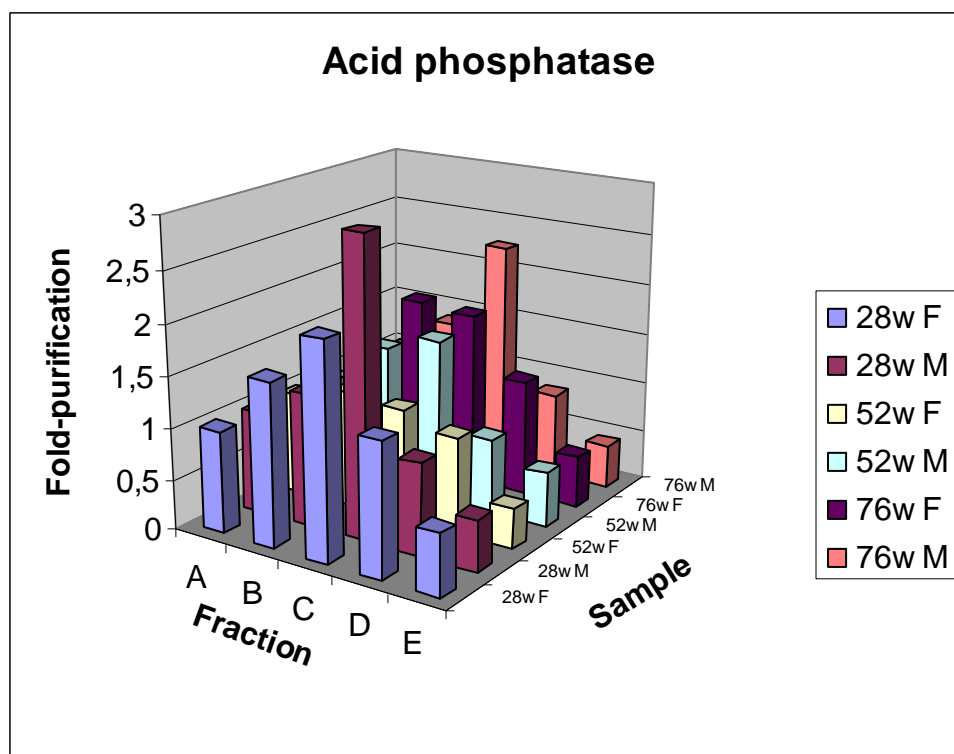

B

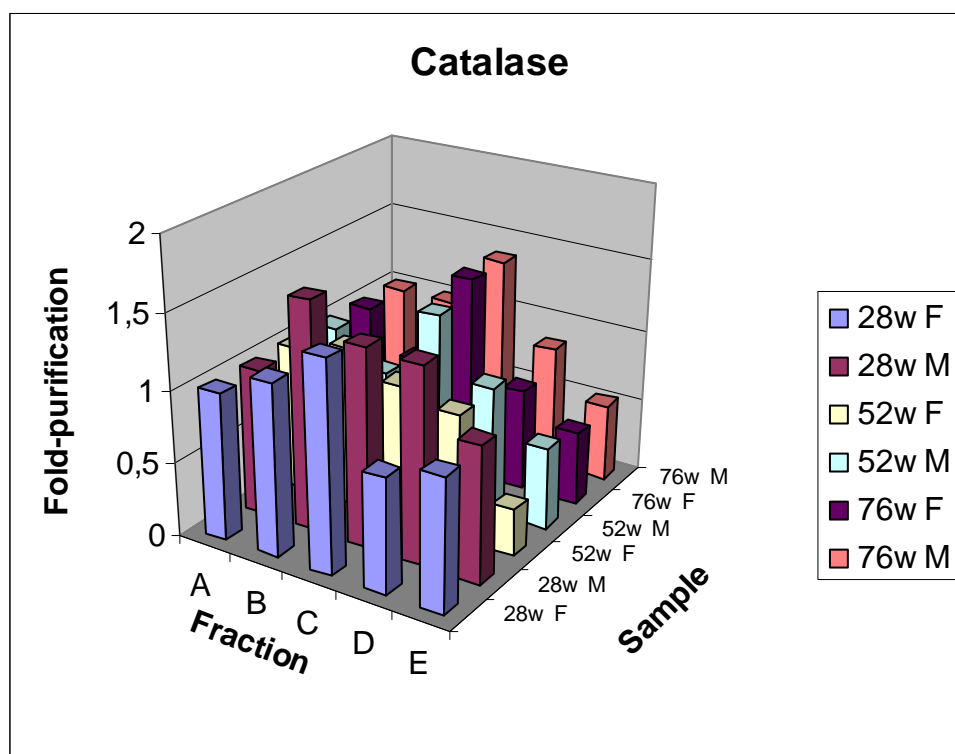

**C**

|   | 28w F | 28w M | 52w F | 52w M | 76w F | 76w M |
|---|-------|-------|-------|-------|-------|-------|
| A | 1     | 1     | 1     | 1     | 1     | 1     |
| B | 1.6   | 1.3   | 1.14  | 1.43  | 1.75  | 1.37  |
| C | 2.1   | 2.9   | 1.1   | 1.6   | 1.7   | 2.25  |
| D | 1.3   | 0.9   | 0.95  | 0.74  | 1.14  | 0.83  |
| E | 0.6   | 0.5   | 0.4   | 0.54  | 0.5   | 0.42  |

**D**

|   | 28w F | 28w M | 52w F | 52w M | 76w F | 76w M |
|---|-------|-------|-------|-------|-------|-------|
| A | 1     | 1     | 1     | 1     | 1     | 1     |
| B | 1.17  | 1.56  | 1.1   | 0.78  | 0.72  | 1     |
| C | 1.43  | 1.35  | 0.94  | 1.27  | 1.38  | 1.37  |
| D | 0.78  | 1.33  | 0.85  | 0.87  | 0.69  | 0.84  |
| E | 0.9   | 0.93  | 0.32  | 0.56  | 0.5   | 0.53  |

Supplement: Additional file 1 — Enrichment of some organellar marker enzymes during the subcellular fractionation procedure. A – Graph illustrating the purification of acid phosphatase (lysosomal marker); B – graph illustrating the purification of catalase (peroxisomal marker); C – supplementary table with information on the absolute values of specific acid phosphatase activity at different fractionation steps; D – supplementary table with information on the absolute values of specific catalase activity at different fractionation steps. A – total homogenate; B – postnuclear fraction; C – heavy mitochondrial fraction; D – organelle – enriched fraction; E – eluate. Purification of an enzyme was calculated as SEA(fr.X)/SEA(fr.A), where SEA is specific enzymatic activity, and fr. X is fraction B – E. [file 1477-5956-7-16-S1.pdf]

Additional file 2

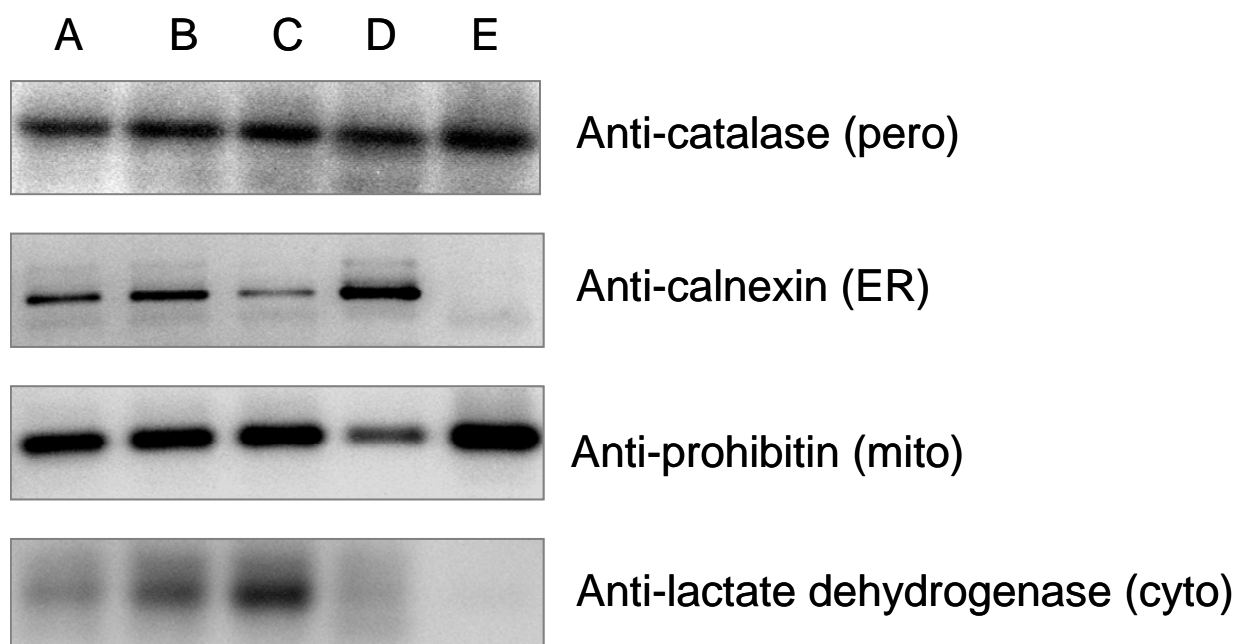

Supplement: Additional file 2 — Representative Western blots illustrating the enrichment of specific organelles during the fractionation procedure. A – total homogenate; B – postnuclear fraction; C – heavy mitochondrial fraction; D – organelle – enriched fraction; E – eluate from LC. [file 1477-5956-7-16-S2.pdf]

# Additional file 3

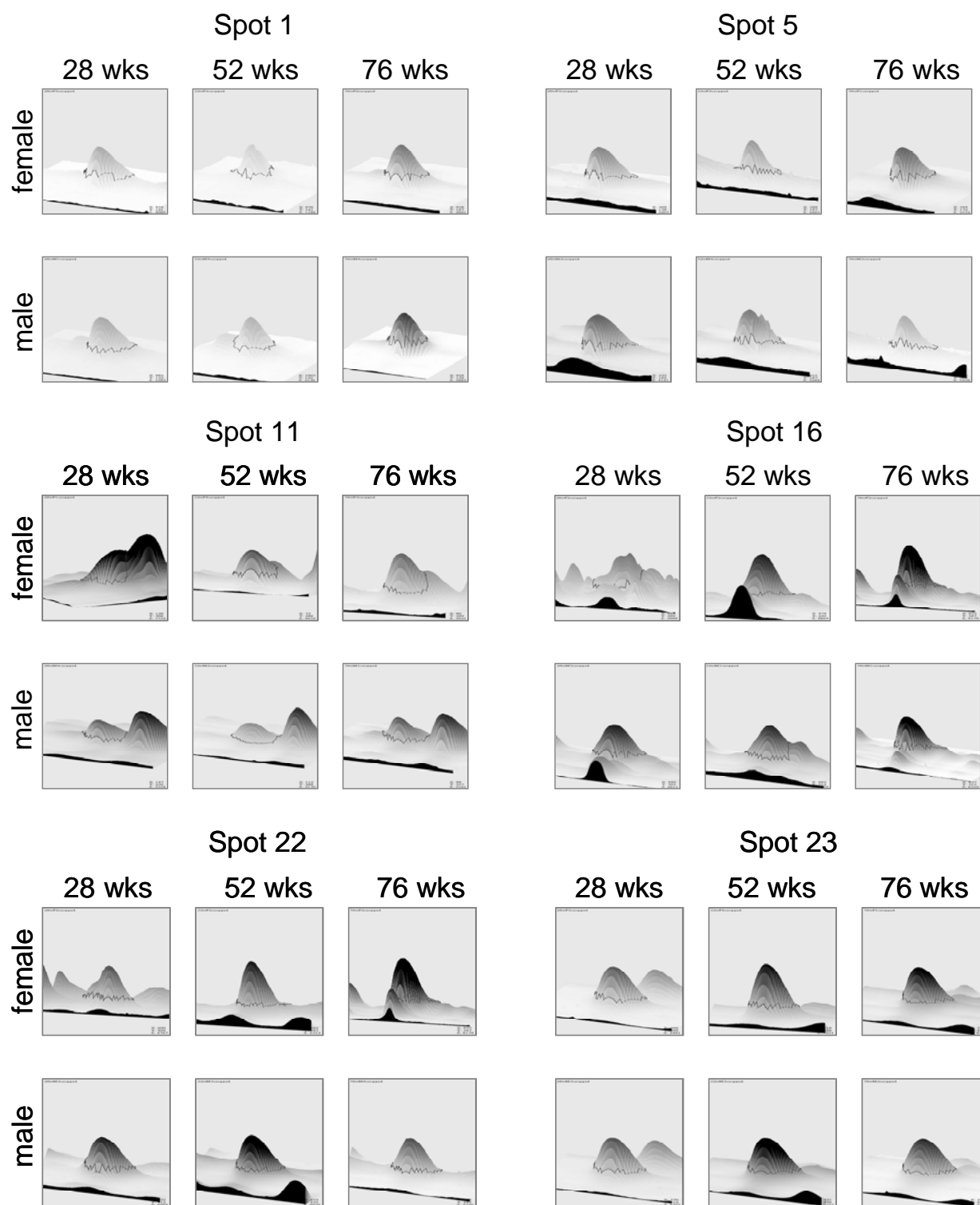

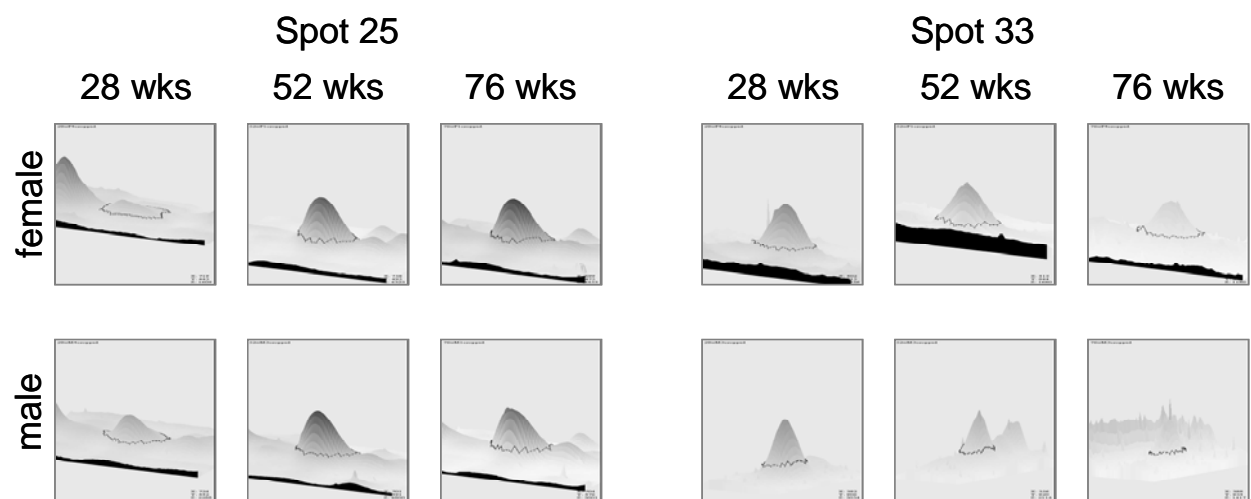

Supplement: Additional file 3 — 3-D views of differentially expressed proteins common for both genders. [file 1477-5956-7-16-S3.pdf]
