# Supplementary material for: Proteomic study on gender differences in aging kidney of mice
Source: Proteome Sci. 2009 Apr 9;7:16. doi: 10.1186/1477-5956-7-16 (PMC2673210; doi:10.1186/1477-5956-7-16)
Supplement: Additional file 4 — Protein expression data. A- Female mouse kidney, B- Male mouse kidney. [file 1477-5956-7-16-S4.pdf]

Additional file 4

A

| Spot no | 28 weeks | | 52 weeks | | 76 weeks | |
|---|---|---|---|---|---|---|
| | Mean | St. Dev. | Mean | St. Dev. | Mean | St. Dev. |
| 1 | 0.249 | 0.053 | 0.219 | 0.050 | 0.383 | 0.027 |
| 2 | 1.156 | 0.188 | 0.685 | 0.185 | 0.619 | 0.210 |
| 3 | 0.260 | 0.134 | 0.611 | 0.193 | 0.362 | 0.122 |
| 4 | 2.179 | 0.600 | 3.159 | 0.473 | 1.512 | 0.373 |
| 5 | 0.579 | 0.120 | 0.313 | 0.037 | 0.595 | 0.022 |
| 6 | 0.338 | 0.129 | 0.171 | 0.029 | 0.680 | 0.107 |
| 7 | 0.309 | 0.042 | 0.358 | 0.074 | 0.880 | 0.156 |
| 8 | 0.231 | 0.038 | 0.327 | 0.045 | 0.141 | 0.045 |
| 9 | 0.478 | 0.077 | 0.663 | 0.152 | 0.810 | 0.104 |
| 10 | 1.903 | 0.261 | 1.923 | 0.215 | 1.251 | 0.298 |
| 11 | 1.367 | 0.139 | 1.017 | 0.503 | 0.639 | 0.252 |
| 12 | 0.306 | 0.114 | 0.136 | 0.037 | 0.994 | 0.110 |
| 13 | 0.550 | 0.121 | 0.378 | 0.174 | 0.208 | 0.023 |
| 14 | 0.626 | 0.078 | 0.497 | 0.150 | 0.282 | 0.045 |
| 15 | 0.143 | 0.050 | 0.192 | 0.062 | 0.406 | 0.083 |
| 16 | 1.546 | 0.324 | 2.224 | 0.272 | 2.482 | 0.196 |
| 17 | 2.234 | 0.701 | 1.576 | 0.143 | 1.175 | 0.320 |
| 18 | 0.612 | 0.156 | 0.875 | 0.223 | 1.125 | 0.190 |
| 19 | 0.181 | 0.039 | 0.121 | 0.015 | 0.111 | 0.018 |
| 20 | 0.056 | 0.017 | 0.077 | 0.025 | 0.117 | 0.021 |
| 21 | 0.323 | 0.047 | 0.254 | 0.015 | 0.139 | 0.013 |
| 22 | 1.097 | 0.147 | 1.391 | 0.177 | 1.610 | 0.151 |
| 23 | 0.699 | 0.193 | 1.036 | 0.130 | 1.236 | 0.108 |
| 24 | 0.372 | 0.023 | 0.309 | 0.060 | 0.578 | 0.086 |
| 25 | 0.357 | 0.115 | 0.695 | 0.080 | 0.758 | 0.068 |
| 26 | 0.302 | 0.099 | 0.275 | 0.107 | 0.530 | 0.091 |
| 27 | 0.239 | 0.027 | 0.201 | 0.027 | 0.110 | 0.011 |
| 28 | 0.241 | 0.076 | 0.352 | 0.087 | 0.415 | 0.080 |
| 29 | 0.160 | 0.039 | 0.168 | 0.025 | 0.229 | 0.034 |
| 30 | 0.100 | 0.041 | 0.139 | 0.024 | 0.206 | 0.052 |
| 31 | 0.132 | 0.013 | 0.178 | 0.024 | 0.146 | 0.017 |
| 32 | 0.219 | 0.056 | 0.433 | 0.064 | 0.544 | 0.056 |
| 33 | 0.287 | 0.041 | 0.324 | 0.031 | 0.193 | 0.071 |

B

| Spot no | 28 weeks | | 52 weeks | | 76 weeks | |
|---|---|---|---|---|---|---|
| | Mean | St. Dev. | Mean | St. Dev. | Mean | St. Dev. |
| 1 | 0.309 | 0.069 | 0.228 | 0.047 | 0.599 | 0.209 |
| 5 | 0.739 | 0.092 | 0.683 | 0.107 | 0.388 | 0.044 |
| 11 | 0.968 | 0.268 | 0.671 | 0.078 | 1.148 | 0.168 |
| 16 | 1.617 | 0.283 | 1.605 | 0.482 | 2.634 | 0.279 |
| 22 | 1.393 | 0.214 | 1.849 | 0.330 | 1.316 | 0.149 |
| 23 | 0.917 | 0.214 | 1.422 | 0.123 | 1.199 | 0.249 |
| 25 | 0.363 | 0.063 | 0.689 | 0.116 | 0.554 | 0.156 |
| 33 | 0.345 | 0.059 | 0.246 | 0.031 | 0.187 | 0.035 |
| 34 | 0.359 | 0.170 | 0.288 | 0.055 | 0.789 | 0.310 |
| 35 | 0.583 | 0.179 | 0.231 | 0.159 | 0.384 | 0.124 |
| 36 | 0.055 | 0.011 | 0.082 | 0.035 | 0.143 | 0.028 |
| 37 | 0.112 | 0.039 | 0.133 | 0.052 | 0.247 | 0.085 |
| 38 | 0.517 | 0.070 | 0.330 | 0.062 | 0.435 | 0.038 |
| 39 | 0.243 | 0.042 | 0.129 | 0.030 | 0.169 | 0.050 |
| 40 | 0.358 | 0.119 | 0.303 | 0.071 | 0.131 | 0.051 |
| 41 | 0.392 | 0.069 | 0.202 | 0.049 | 0.222 | 0.117 |
| 42 | 0.081 | 0.024 | 0.091 | 0.029 | 0.132 | 0.016 |
| 43 | 1.340 | 0.459 | 0.950 | 0.175 | 1.614 | 0.267 |
| 44 | 0.116 | 0.011 | 0.121 | 0.025 | 0.174 | 0.027 |
| 45 | 0.096 | 0.009 | 0.108 | 0.004 | 0.124 | 0.012 |
| 46 | 0.616 | 0.175 | 0.861 | 0.042 | 0.943 | 0.121 |
| 47 | 0.349 | 0.069 | 0.327 | 0.052 | 0.214 | 0.059 |
