# Supplementary material for: Proteomic study on gender differences in aging kidney of mice
Source: Proteome Sci. 2009 Apr 9;7:16. doi: 10.1186/1477-5956-7-16 (PMC2673210; doi:10.1186/1477-5956-7-16)
Supplement: Additional file 5 — List of age-related mouse kidney proteins identified by MALDI-TOF-MS. (A) Female; (B) Male. Proteins' numbers correspond to the numbers from 2-DE gels shown in Figs. 1 and 2. Obs., observed. [file 1477-5956-7-16-S5.pdf]

Additional file 5

A

Ref Nr	Protein name	52/28 wks	76/28 wks	Function	Localization	NCBI nr	Coverage, %	No of matched peaks	Score	p	Mr theor	Mr obs	pI
1	transferrin	-1,14	1,53	ion transporter	extracell	gi 20330802	47	30/69	241	9,00E-20	76724	76700	7,2
2	78 kDa glucose-regulated protein	-1,69	-1,78	chaperone	ER	gi 2506545	48	27/68	209	1,40E-16	72422	72400	5,2
3	heat shock protein 9A	2,35	1,39	chaperone	mito	gi 162461907	44	26/67	184	4,50E-14	73461	71500	5,8-5,9
4	albumin	1,45	-1,44	transporter	ER	gi 163310765	38	20/46	176	2,80E-13	68693	65700	6
5	heat shock protein 9A	-1,85	1,03	chaperone	mito	gi 162461907	27	16/75	72	0,0078	73461	66600	6,3
6	Hspd1 protein	-1,98	2	chaperone	mito	gi 76779273	40	18/82	106	2,80E-06	60955	61200	5,7
7	leucine aminopeptidase	1,16	2,85	exopeptidase	cyt	gi 124028616	57	32/57	268	1,80E-22	56141	57100	7,4
8	glutamate dehydrogenase 1	1,42	-1,64	glutamate catabolism	mito	gi 148692928	43	21/80	111	1,10E-06	54219	54700	7,6
9	aldehyde dehydrogenase	1,39	1,7	aldehydes' oxidation	mito	gi 6753036	38	17/55	138	1,80E-09	56538	53200	6,9
10	ATP syntase, beta subunit	1,01	-1,52	ATP synthesis/hydrolysis	mito	gi 89574015	62	23/78	153	5,70E-11	56300	52500	5,2
11	ATP synthase, F1 complex	-1,34	-2,14	ATP synthesis/hydrolysis	mito	gi 31980648	39	23/103	90	0,00011	56300	52500	5,1
12	D-lactate dehydrogenase	-2,25	3,25	FAD binding domain	mito	gi 34328379	51	21/128	89	0,00015	51848	52500	6,4
15	ornithine aminotransferase	1,34	2,84	amino acid metabolism	mito	gi 8393866	41	16/59	122	3,10E-06	48355	47500	6,3
16	isocitrate dehydrogenase 1	1,44	1,6	carbohydrate metabolism	cyt	gi 74144459	67	33/275	94	5,10E-05	46674	45000	7,2
17	gamma-actin	-3,19	-1,9	cytoskeleton	cyt	gi 809561	52	17/52	108	1,80E-06	41793	45000	5,5
18	isovaleryl coenzyme A dehydrogenase	1,43	1,84	β-oxidation	mito	gi 9789985	32	13/52	95	3,50E-05	46325	45000	7
20	isocitrate dehydrogenase 3	1,38	2,1	carbohydrate metabolism	mito	gi 18250284	39	33/275	87	0,00024	39693	39800	5,8
21	fructose bisphosphatase 1	-1,27	-2,33	carbohydrate metabolism	cyt	gi 9506589	61	19/37	192	7,10E-15	36912	38500	6,4-6,5
22	hypothetical protein LOC70984	1,27	1,47	unknown (hydrolase?)	cyt	gi 19526926	45	14/58	75	0,0033	34996	33000	6,4
23	hypothetical protein LOC70984	1,48	1,77	unknown (hydrolase?)	cyt	gi 19526926	22	8/23	105	3,60E-06	34996	32500	6,3
24	lactamase, beta 2	-1,21	1,55	glyoxalytic activity	mito	gi 21703764	34	9/37	77	0,0025	32754	29100	6,5
25	3-hydroxyisobutyrate dehydrogenase	1,95	2,12	oxidative carboxylase	mito	gi 21704140	34	12/54	70	0,012	35440	28300	6,6
26	GRIN1	-1,1	1,75	neurite outgrowth induction	cyt	gi 5901688	30	25/60	72	0,0078	95496	26000	5,3
27	phosphoglycerate mutase 1	-1,19	-2,18	transport and metabolism	cyt/lyso	gi 114326546	56	12/72	80	0,0012	28832	26000	7,9

B

Ref Nr	Protein name	52/28 wks	76/28 wks	Function	Localization	NCBI nr	Coverage, %	No of matched peaks	Score	p	Mr theor	Mr obs	pI
1	transferrin	-1,36	1,94	iron transporter	extracell	gi 20330802	42	26/74	194	4,50E-15	76724	76700	7,2-7,3
34	transferrin	-1,25	2,2	iron transporter	extracell	gi 20330802	24	14/36	111	9,00E-07	76724	76700	7,4
5	heat shock protein 9A	-1,1	-1,9	chaperone	mito	gi 162461907	67	45/204	175	3,60E-13	73461	66600	6,3
35	catalase	-2,52	-1,52	antioxidant	pero	gi 115704	45	23/59	189	1,40E-14	59765	57100	7,6
36	aldehyde dehydrogenase 6, A1	1,5	2,61	aldehydes' oxidation	mito	gi 19527258	35	14/76	83	0,00055	57916	54700	7,6
37	glutathione synthetase	1,19	2,21	glutathione biosynthesis	cyt	gi 6680117	37	18/56	154	4,50E-11	52247	54700	5,7-5,8
11	ATP synthase, F1 complex	-1,44	1,19	ATP synthesis/hydrolysis	mito	gi 31980648	39	23/103	90	0,00011	56300	52500	5,2
16	isocitrate dehydrogenase 1	-1,01	1,63	carbohydrate metabolism	cyto	gi 74144459	67	33/275	94	5,10E-05	46674	45000	7,2
39	phosphotriesterase related	-1,88	-1,44	organophosphates' detoxification	cyt/mito	gi 6679525	50	15/98	100	1,20E-05	39218	41300	7
41	aldo-keto reductase 1, A4	-1,94	-1,77	oxidoreductase	cyt/mito	gi 10946870	49	15/51	146	2,80E-10	36587	38500	7,7-7,8
22	hypothetical protein LOC70984	1,33	-1,1	unknown (hydrolase?)	cyt	gi 19526926	22	8/23	75	0,0033	34996	33000	6,4
23	hypothetical protein LOC70984	1,55	1,31	unknown (hydrolase?)	cyt	gi 19526926	45	14/58	105	3,60E-06	34996	32500	6,3
25	3-hydroxyisobutyrate dehydrogenase	1,9	1,51	oxidative carboxylase	mito	gi 21704140	38	11/54	82	0,0007	35440	28300	6,7
46	peroxiredoxin-1	1,4	1,53	thiol-specific antioxidant	cyt	gi 123230136	75	18/87	153	6,90E-11	18927	19300	6,6-6,7
